# The Certificate of the Cause of Death

**Published:** 1916-06-17

**Authors:** 


### THE CERTIFICATE OF THE CAUSE OF DEATH.

THE official certificate of the cause of death has been the subject of many detailed criticisms, and when in happier times the Legislature finds opportunity for the consideration of social questions some of these criticisms will probably be recognised as valid. At the outset is the fact that the certificate is not a certain guarantee that the death of the individual to whom it refers has actually taken place. The practitioner testifies that he has been in attendance on a patient for a given illness, and that he has been "informed" that death has occurred, but he is not compelled to give personal testimony to the truth of this information; and instances have occurred in which the certificate in this respect has been proved to be incorrect. In regard to this it is morally certain there will be amendment. According to the existing law the practitioner receives no fee for his certificate, but if, as is likely, he is compelled personally to verify the fact of death, it would be very unjust to demand this further service without recognition. In not a few instances it would mean, not only expert knowledge and responsibility but also a special journey, and sometimes a long journey. The profession will have to be alert on this point seeing that our legislators have recently decreed that the certifying of a diagnosis of infectious disease is little more than clerical duty. Such a conclusion may prove a precedent of evil omen.

A second point which has excited criticism is the handing over of the certificate to one of the relatives of the deceased. Undoubtedly this does not make for candour and accuracy in the certificate. Social and even humanitarian feelings prompt to obscurity and to a veiling of the truth when a full revelation would cause pain or anxiety to the survivors, and this factor does undoubtedly minimise the value of the Registrar-General's statistical statement. It has been suggested that the certificate should be treated as a confidential document, and should be delivered to the Registrar, who would be forbidden to give the full particulars except to some duly authorised person. Such an arrangement would, of course, be quite acceptable to the medical profession, though even here there would be some sense of disadvantage. Possibly nowadays a family history does not occupy so prominent a place in clinical records as was the case some years ago, yet it still has some significance, and if it is to be considered at all it ought to be full and accurate. The concealment of the exact cause of death from the public would in some degree interfere with the family record. A more serious objection would probably come from the public, who would certainly dislike any appearance of mystery and concealment about the nature of the fatal illness. Such an attitude can hardly fail to excite a sympathy which a plea for accurate statistics would hardly assuage. The only way of escape would seem to be the establishment of two certificates, one stating the nature of the last illness in general terms suitable for the contemplation of bereaved relatives, and a second giving the more precise pathological particulars demanded by scientific classification. The latter are of importance to the ordering of the health of the nation, but if they are to be obtained the process must not involve the infliction of needless pain on those who are already suffering under a sense of grief and loss.

